# Real-world treatment patterns and clinical burden of patients with hereditary angioedema treated with long-term prophylaxis

**DOI:** 10.1186/s13023-026-04391-6

**Published:** 2026-05-19

**Authors:** William Lumry, Art Zbrozek, Philippe Thompson-Leduc, Montserrat Vera-Llonch, Marjolaine Gauthier-Loiselle, Claire Vanden Eynde, Anabelle Tardif-Samson, Annie Guérin, Cristine Radojicic

**Affiliations:** 1 AARA Research Center, Dallas, TX USA; 2 Ionis Pharmaceuticals, Carlsbad, CA USA; 3 Analysis Group, Inc, Montreal, QC Canada 1190 avenue des Canadiens-de-Montréal, Tour Deloitte, Suite 1500, H3B 0G7; 4 Duke University School of Medicine, Durham, NC USA

## Abstract

**Background:**

Long-term prophylaxis (LTP) may reduce the hereditary angioedema (HAE) burden by reducing attack frequency, but real-world evidence is limited.

**Objective:**

To describe LTP treatment patterns and the frequency of HAE attacks leading to emergency department or inpatient visits (ED/IP) and healthcare resource use (HRU) before and after LTP initiation.

**Methods:**

Patients initiating LTP in the Komodo Research Database (1/1/2021-1/30/2024) were followed until discontinuation or data end. Treatment adherence overall and among patients with > 30 days of treatment was measured as the proportion of days covered ≥ 80%. Treatment persistence, defined as no interruption ≥ 90 days or switch, was evaluated overall and among patients with > 30 days of treatment using Kaplan-Meier (KM) analysis with KM rates and corresponding 95% confidence intervals (CI). Rates of HAE attacks leading to ED/IP and per-patient-per-year all-cause HRU before and after LTP initiation were reported among patients with > 30 days of treatment. All analyses were descriptive and no statistical testing was conducted.

**Results:**

Overall, 499 patients were included (57 Haegarda [11%], 257 Orladeyo [52%], 185 Takhzyro [37%]; mean follow-up = 11.3 months; median age = 39 years; 69% female), among whom 449 had > 30 days of treatment (mean follow-up = 12.5 months). At 12 months after LTP initiation, 52.6% of patients in the overall study sample and 57.0% of patients with > 30 days of treatment were adherent. Based on KM analysis, persistence rates at 12 months after LTP initiation were 58.7% (95% CI = 53.8%; 63.4%) in the overall sample and 65.1% (59.9%; 69.9%) among patients with > 30 days of treatment. Among the patients with > 30 days of treatment, 38% had ≥ 1 HAE attack leading to ED/IP in the year before LTP initiation (mean = 0.82 attacks), decreasing to 23% after (mean = 0.51 attacks). In these 449 patients, annual HRU remained substantial before and after LTP initiation across settings (IP = 2.4 and 1.7 days [0.3 and 0.2 admissions]; ED = 1.5 and 1.2 visits; outpatient = 21.0 and 19.3 visits, respectively).

**Conclusion:**

Although recently available LTP can partly reduce the clinical burden of patients with HAE, novel therapies are needed to further alleviate this burden.

## Introduction

Hereditary angioedema (HAE) is a rare autosomal dominant condition commonly caused by dysfunction in the C1 esterase inhibitor (C1-INH) which affects approximately 1 in 50,000 individuals in the United States (US) [1]. The insufficient/non-functional C1-INH fails to regulate the factor XII-kallikrein cascade responsible for the generation of the pro-inflammatory peptide, bradykinin, which in turns increases vascular permeability and leads to angioedema [2]. HAE symptoms, which include swelling of the skin and subcutaneous tissues, gastrointestinal tract (which can result in debilitating abdominal pain), or upper airways (including laryngeal edema which can be life-threatening), tend to manifest later in childhood and are similar in adult and pediatric patients [3,4].

HAE is characterized by recurrent attacks which can vary widely in frequency and duration [3, 4]. A survey of patients with HAE recruited via the US Hereditary Angioedema Association reported between 0 and 350 HAE attacks per patient per year (PPPY) which ranged in duration from 1 h to 15 days [5]. Moreover, one survey study of US physicians reported that although approximately 47% of HAE attacks are adequately managed with medication at home, 35% of attacks are more severe, requiring an emergency department (ED) visit [4]. In a multicenter prospective study, 30% of HAE attacks treated in the ED eventually led to an inpatient (IP) admission [6]. As such, HAE attacks can lead to a significant clinical and economic burden, as well as meaningfully impact the patients’ quality of life (QoL) [7–9].

Several on-demand treatment (ODT) injectable options have proven effective in stopping the progression of HAE attacks once they occur, including human plasma-derived C1-INH (Berinert^®^; approved by the US Food and Drug Administration [FDA] in 2009), ecallantide (Kalbitor^®^; approved in 2009), recombinant C1-INH (Ruconest^®^; approved in 2014), icatibant (Firazyr^®^; approved in 2011), and branded-generic icatibant (Sajazir™; approved in 2021). In July 2025, the US FDA also approved sebetralstat (Ekterly^®^), an oral ODT formulation that may lead to a more timely treatment of HAE attacks than parenteral options [10].

There are also several long-term prophylactic (LTP) agents approved by the FDA for the prevention of HAE attacks. In particular, in the past decade, three new LTP options have been approved by the FDA based on their efficacy in reducing the risk and/or incidence of HAE attacks [11]: human C1-INH (Haegarda^®^; approved in 2017) which replaces the insufficient/non-functional C1-INH, and lanadelumab (Takhzyro^®^; approved in 2018) and berotralstat (Orladeyo^®^; approved in 2020) which both work to inhibit the active plasma kallikrein. In addition, the FDA recently approved garadacimab (Andembry^®^, which inhibits activated factor XII) and donidalorsen (Dawnzera™, which reduces pre-kallikrein levels) for the prevention of HAE attacks in June and August 2025, respectively [12, 13].

Given the evolving treatment landscape, additional evidence is needed to understand the current clinical burden of HAE, as well as the use and effectiveness of LTP treatment in the real world. To that end, this study aimed to describe treatment adherence and persistence to commonly used LTP agents (i.e., human C1-INH, lanadelumab, and berotralstat), the frequency of HAE attacks leading to an ED or IP visit (ED/IP), and the all-cause healthcare resource utilization (HRU) before and after LTP initiation in a real-world US setting.

## Methods

### Data source

Administrative claims data between January 1, 2016 and February, 29, 2024 from the Komodo Research Database (KRD) were used. The KRD captures a large, diverse patient cohort and ensures socioeconomic diversity by sourcing closed claims data directly from over 150 commercial, Medicare Advantage, Medicaid, Managed Medicaid, and other (i.e., Tricare, workers comp) payers representing more than 170 million patient lives. The database captures IP and outpatient (OP) diagnoses and procedures, pharmacy fills, payer type and name, mortality data, and demographic data of US patients of all ages, incomes, races, and ethnicities. The data are de-identified and comply with the Health Insurance Portability and Accountability Act (HIPAA) regulations. The study was considered exempt research under HIPAA. Therefore, no Institutional Review Board or Ethics Committee approval was sought for the conduct of the study.

### Study design and sample selection

**Fig. 1:**
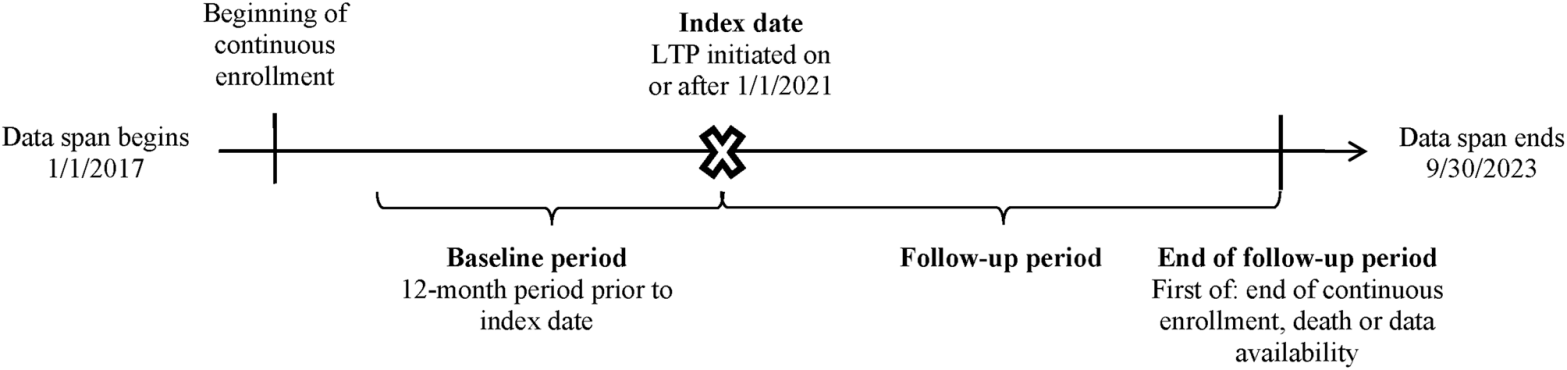
Study design. Abbreviation: LTP = long-term prophylaxis

A retrospective cohort study design was used (Fig. 1). Since there are no specific International Classification of Diseases, Tenth Revision, Clinical Modification (ICD-10-CM) diagnostic codes for HAE, patients were defined as those who initiated an LTP of interest (i.e., human C1-INH, lanadelumab, or berotralstat) based on ≥ 1 pharmacy prescription fill using their respective National Drug Codes. Garadacimab and donidalorsen were not considered as they were approved after the end of the data period. Patients were required to have ≥ 1 pharmacy prescription fill for an LTP, to avoid the inclusion of patients who received an LTP in a medical setting exclusively, which may not reflect long-term use of prophylactic treatment. The index date was defined as the date of LTP initiation (i.e., the first recorded administration or pharmacy fill for an LTP) on or after January 1, 2021 (to restrict the analyses to a calendar period during which all three LTP of interest were FDA-approved and available on the market). Patients were excluded from the study if they initiated more than one LTP of interest on or within 30 days of the index date. Finally, to be included in the study, patients were required to be ≥ 12 years old as of the index date, have ≥ 12 months of continuous insurance enrollment prior to the index date and ≥ 30 days of continuous insurance enrollment following the index date. The baseline period spanned the 12 months prior to the index date and the follow-up period spanned from the index date until the earliest of the end of continuous insurance enrollment, death, or end of data availability.

### Study measures and outcomes

Patient demographics (i.e., age at the index date, sex, race/ethnicity, region, and plan type) and clinical characteristics (i.e., Quan-Charlson Comorbidity Index, [14] comorbidities, and use of prior LTP) were described during the baseline period or on the index date.

Adherence to treatment was measured using the proportion of days covered (PDC), which is the ratio of unique days during which the patient had the treatment of interest “on hand” (i.e., days of administrations/prescription fills + days of supply) over a fixed period of time. The PDC was evaluated at 6 and 12 months after the index date among patients with continuous health plan enrollment for the corresponding period. Patients with PDC ≥ 80% were considered adherent to treatment.

Treatment persistence was measured as the time on treatment from the index date until the earliest of treatment discontinuation or switch. Treatment discontinuation was defined as a gap of ≥ 90 days without the index treatment on hand and the date of discontinuation was defined as the date of the last day of supply. Treatment switch was defined as a prescription fill for a different LTP than the index treatment, and the date of switch was defined as the date of such prescription fill.

HAE attacks leading to ED/IP were identified based on an ED visit or IP admission with a treatment for HAE or evidence of one of the following diagnoses: deficiency in C1-INH system, angioneurotic edema, respiratory failure, dyspnea, stridor, abdominal and pelvic pain, nausea and vomiting, intra-abdominal and pelvic swelling, or localized swelling. All-cause HRU was defined by setting of care, and comprised the number of IP admissions, IP days, ED visits, and OP visits. The rate of HAE attacks leading to ED/IP and the all-cause HRU were reported during the baseline period and during treatment (i.e., between the index date and treatment discontinuation, switch, or end of the follow-up period, whichever occurred first).

### Statistical analyses

Means, standard deviations, and medians were used to describe continuous variables and frequencies and proportions were used to describe categorical variables. PDC at 6 and 12 months after the index date was calculated in the overall study sample and separately among patients with > 30 days of uninterrupted treatment. Treatment persistence was described in the overall study sample and separately among patients with > 30 days of uninterrupted treatment using Kaplan-Meier (KM) analysis with KM rates and 95% confidence intervals (CI) reported at 3, 6, 12, and 24 months post index. Patients who did not discontinue or switch their index LTP were censored at the end of their follow-up period.

The frequency of HAE attacks leading to ED/IP and all-cause HRU were described PPPY among patients with > 30 days of uninterrupted treatment to ensure that the patients were treated for a sufficient duration to experience the therapeutic effect of their index treatment (i.e., to exclude patients who received only short-term or one-time treatment).

All analyses were descriptive and no statistical testing was conducted. Analyses were conducted using SAS^®^ Enterprise Guide^®^ version 7.1 (SAS Institute, Cary, NC, USA).

## Results

### Study sample

**Fig. 2:**
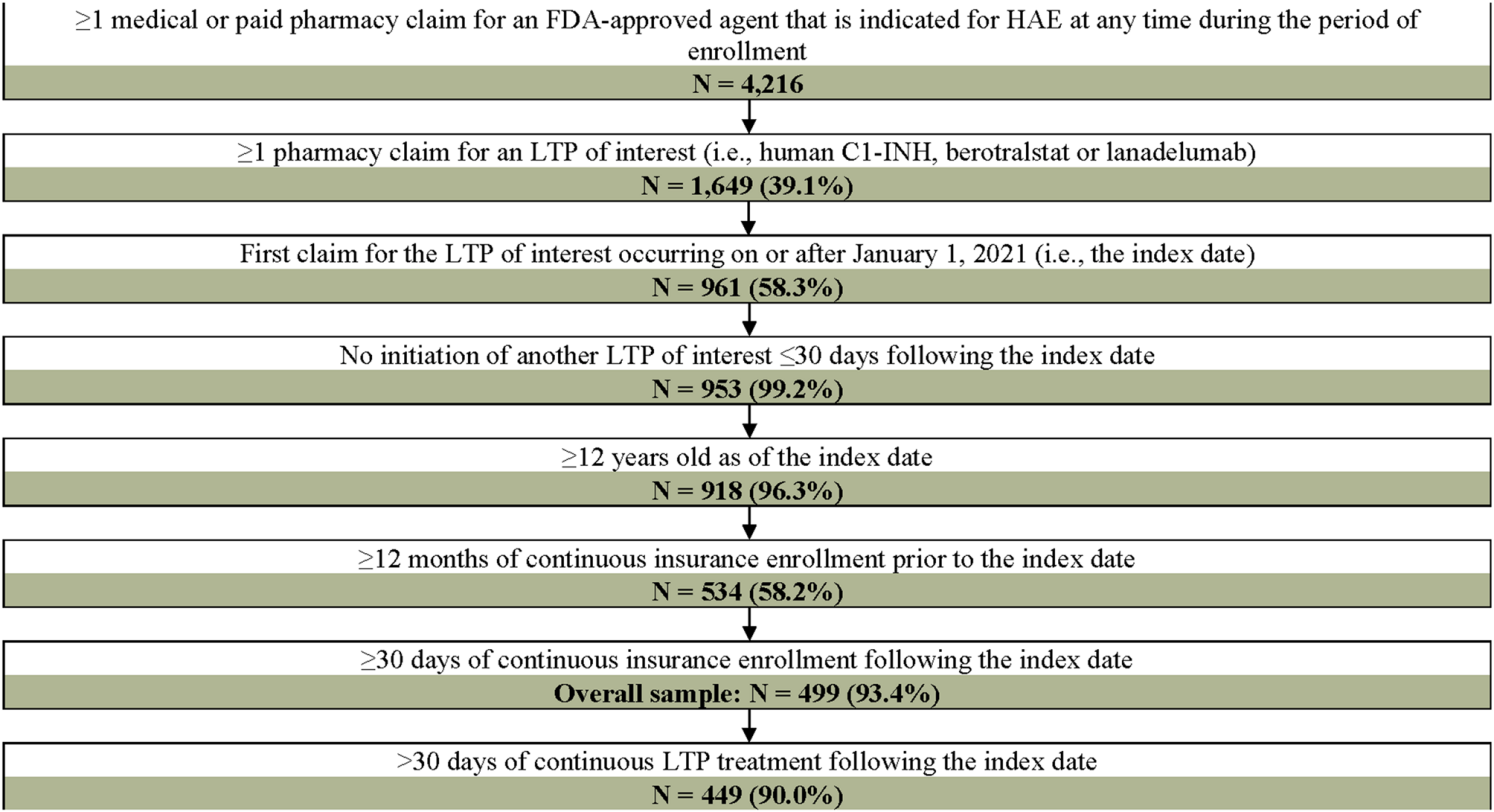
Sample selection. Abbreviations: C1-INH = C1 esterase inhibitor; FDA = Food and Drug Administration; HAE = hereditary angioedema; LTP = long-term prophylaxis

A total of 499 patients were included in the study (Fig. 2, the overall study sample) with a mean (median) age of 40.4 (39.0) years and 69.3% females (Table 1). Common comorbidities included metabolic disorder (32.5%), obesity (29.1%), and chronic pulmonary disease (23.0%). Berotralstat was the most frequently initiated LTP on the index date (51.5%) followed by lanadelumab (37.1%) and human C1-INH (11.4%); 13.8% of patients were treated with a different LTP prior to the index date. The mean (median) follow-up duration was 18.0 (16.9) months.

**Fig. 3:**
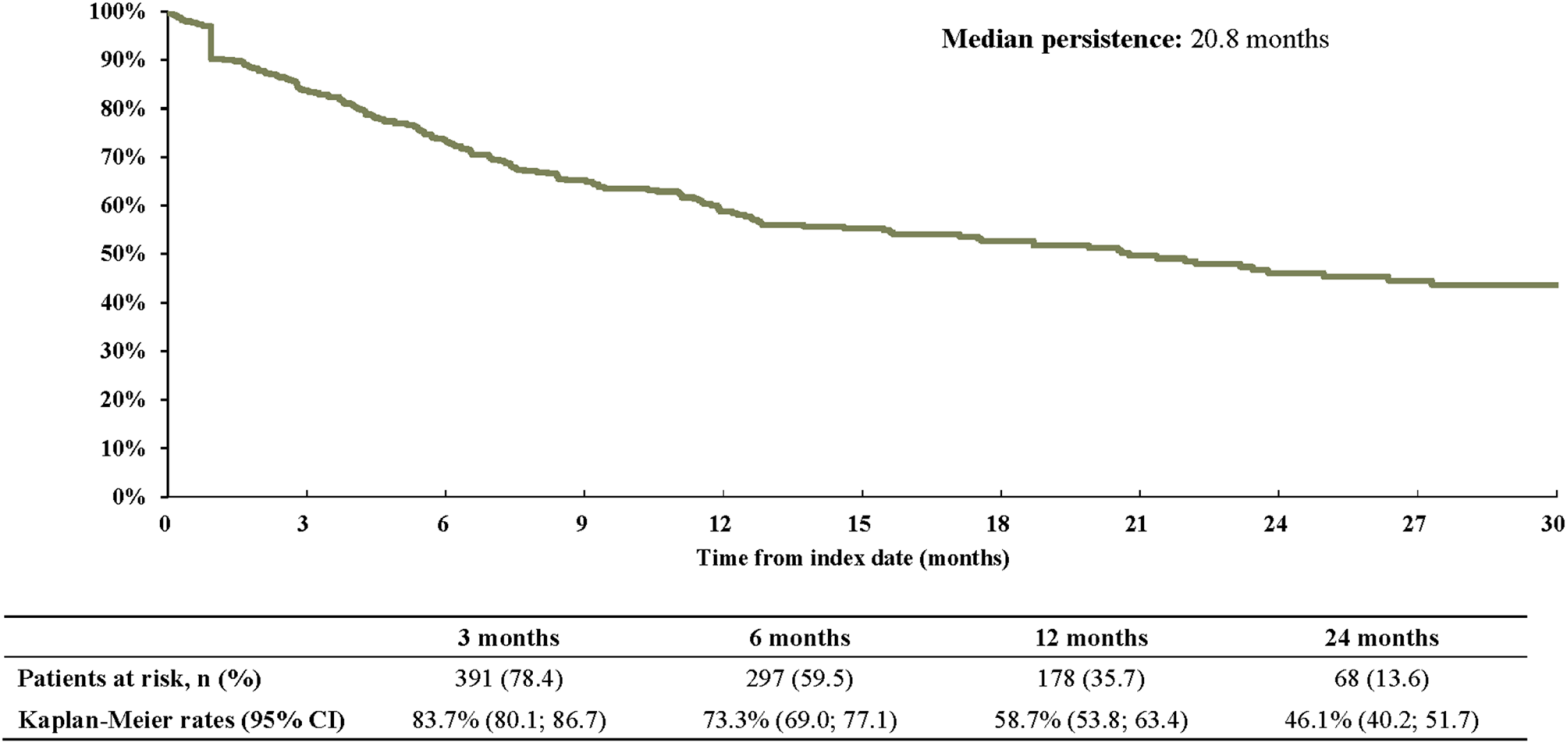
Kaplan-Meier analysis of persistence on index treatment in the overall study sample. Abbreviation: CI = confidence interval

**Fig. 4:**
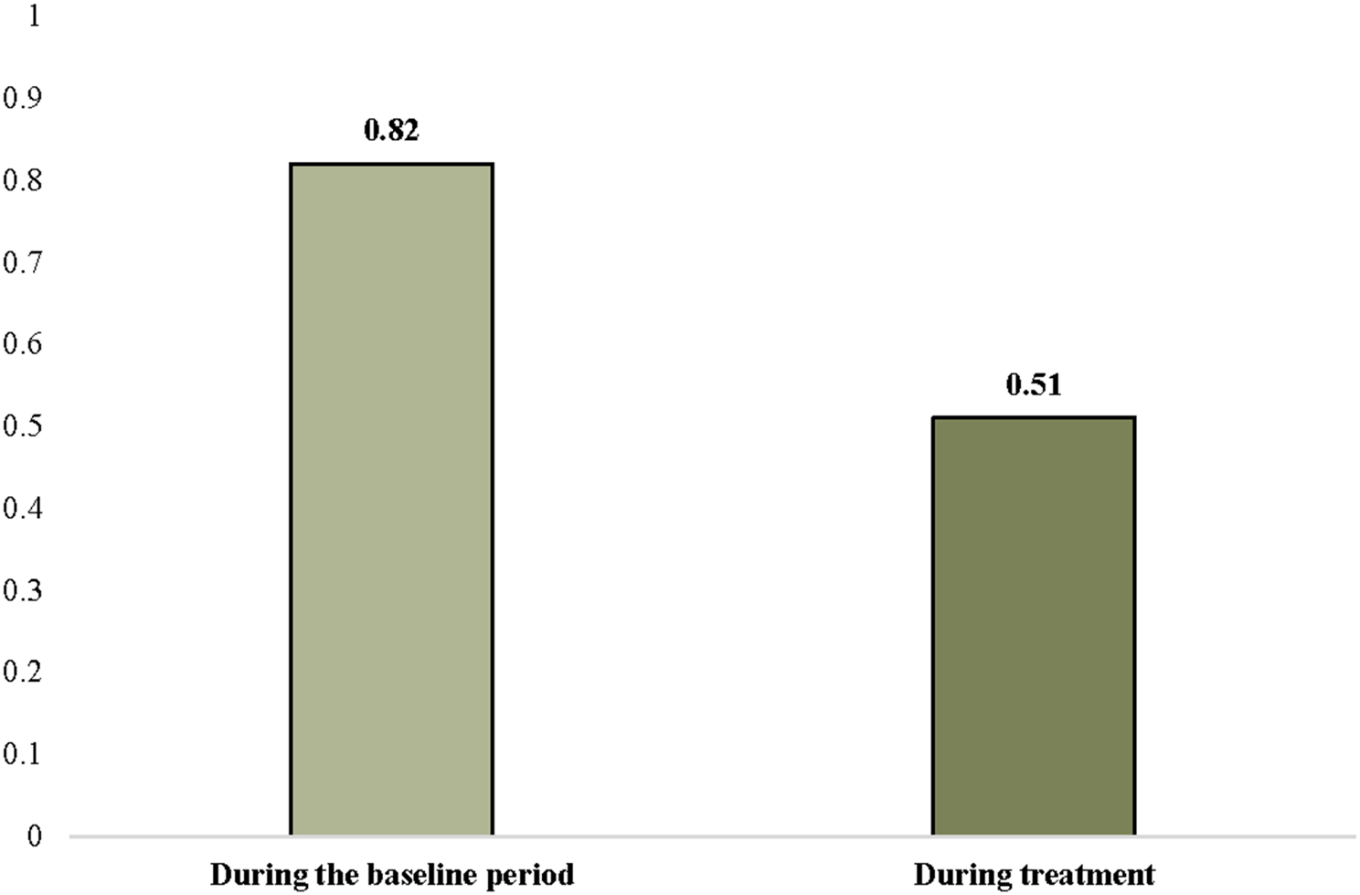
Mean annual number of HAE attacks leading to ED/IP before and after LTP initiation^1^. Abbreviations: C1-INH = C1 esterase inhibitor; ED = emergency department; IP = inpatient; HAE = hereditary angioedema; LTP = long-term prophylaxis. Note: [1] Identified as an ED visit or IP admission with a treatment for HAE or evidence of one of the following diagnoses: deficiency in C1-INH system, angioneurotic edema, respiratory failure, dyspnea, stridor, abdominal and pelvic pain, nausea and vomiting, intra-abdominal and pelvic swelling or localized swelling

**Fig. 5:**
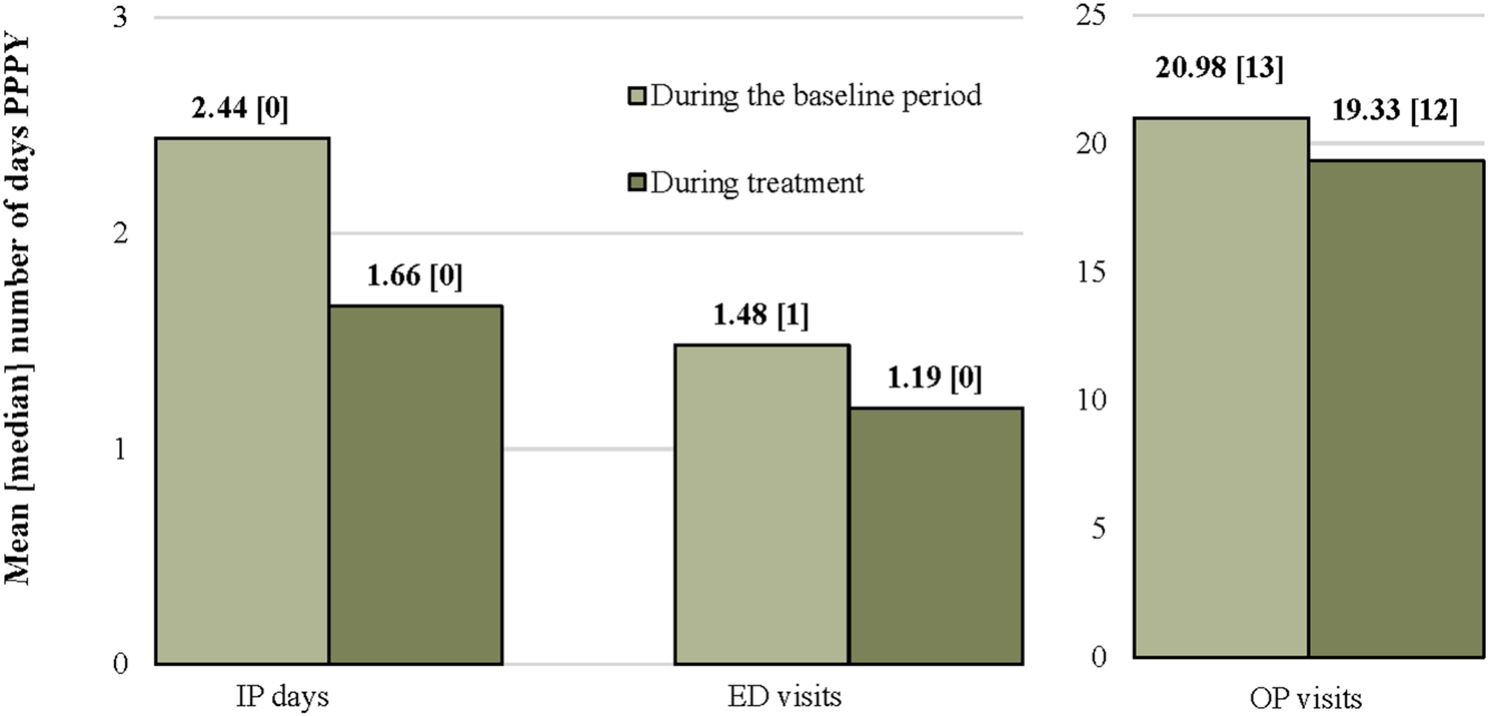
Mean annual rate of all-cause HRU before and after LTP initiation. Abbreviations: ED = emergency department; HRU = healthcare resource use; IP = inpatient; LTP = long-term prophylaxis; OP = outpatient; PPPY = per patient per year

Of these 499 patients, 449 (90.0%) had > 30 days of continuous LTP treatment following the index date.

**Table 1:** Patient demographic and clinical characteristics

	Patients with HAE treated with LTP *N* = 499
Age at the index date, mean ± SD [median]	40.4 ± 16.4 [39.0]
Sex, *n* (%)	
Female	346 (69.3)
Male	149 (29.9)
Unknown	4 (0.8)
**Race/ethnicity**,** n (%)**	
White	244 (48.9)
Black or African-American	47 (9.4)
Hispanic or Latino	46 (9.2)
Other	22 (4.4)
**Region at the index date**,** n (%)**	
South	201 (40.3)
Northeast	111 (22.2)
West	100 (20.0)
Midwest	87 (17.4)
**Plan type at the index date**,** n (%)**	
Commercial	379 (76.0)
Medicaid	65 (13.0)
Medicare Advantage	55 (11.0)
**Quan-CCI**^**1**^, **mean ± SD [median]**	**0.7 ± 1.3 [0.0]**
**Comorbidities of interest**^**1**^, **n (%)**	
Metabolic disorder	162 (32.5)
Obesity	145 (29.1)
Immunosuppressive conditions/treatments^2^	44 (8.8)
**Any use of prior LTP**^**1**^, **n (%)**	**69 (13.8)**
Human C1-INH	39 (7.8)
Berotralstat	0 (0.0)
Lanadelumab	33 (6.6)

Abbreviations: C1-INH = C1 esterase inhibitor; CCI = Charlson Comorbidity Index; HAE = hereditary angioedema; LTP = long-term prophylaxis; SD = standard deviation

Notes: [1] Evaluated during the 12-month baseline period. [2] Included evidence of neoplasm, encounters for radiation/therapy/chemotherapy/immunotherapy, or transplanted organ and tissue status

### Treatment patterns

In the overall study sample, the mean (median) number of prescription fills for the index treatment was 12.4 (8) with 17.6% of patients having only 1 or 2 prescription fills (Table 2). At 6 months post index, 61.1% of the patients were adherent to their index treatment (i.e., PDC ≥ 80%) and the mean (median) PDC was 75.0% (87.8%). At 12 months post index, 52.6% of the patients were adherent to their index treatment and the mean (median) PDC was 66.7% (82.5%). The proportions of patients who discontinued or switched their index LTP were 30.5% and 11.2%, respectively.

Based on the Kaplan-Meier analysis, median persistence in the overall study sample was 20.8 months (Fig. 3). The persistence rates (95% CI) at 12 and 24 months were 58.7% (53.8%; 63.4%) and 46.1% (40.2%; 51.7%), respectively.

Among the subset of patients with > 30 days of treatment (*n* = 449), the mean (median) number of prescription fills for the index treatment was 13.6 (10.0; Table 2). At 6 months post index, 68.4% of the patients were adherent to their index treatment and the mean (median) PDC was 82.1% (91.2%). At 12 months post index, 57.0% of the patients were adherent to their index treatment and the mean (median) PDC was 71.6% (85.0%). The proportions of patients who discontinued or switched their index LTP were 26.1% and 9.4%, respectively.

Median persistence in the subset of patients with > 30 days of treatment was 26.4 months, and the persistence rates (95% CI) at 12 and 24 months were 65.1% (59.9%; 69.9%) and 51.1% (44.7%; 57.1%), respectively.

**Table 2:** Treatment patterns

	Patients with HAE treated with LTP *N* = 499	Patients with HAE treated with LTP for > 30 days *N* = 449
**Duration of post-index data availability (months), mean ± SD [median]**	18.0 ± 10.9 [16.9]	18.2 ± 10.9 [17.5]
**Duration of index treatment (months)**,** mean ± SD [median]**	11.3 ± 9.9 [8.1]	12.5 ± 9.8 [9.3]
**Number of observed prescription fills**,** mean ± SD [median]**	12.4 ± 12.0 [8]	13.6 ± 12.0 [10]
**Adherence**		
≥ 6 months of continuous health plan enrollment, n (%)	416 (83.4)	372 (82.9)
PDC at 6 months (%), mean ± SD [median]	75.0 ± 28.4 [87.8]	82.1 ± 20.8 [91.2]
≥ 50%, n (%)	320 (77.3)	320 (86.5)
≥ 80%, n (%)	253 (61.1)	253 (68.4)
≥ 12 months of continuous health plan enrollment, n (%)	311 (62.3)	287 (63.9)
PDC at 12 months (%), mean ± SD [median]	66.7 ± 31.8 [82.5]	71.6 ± 27.9 [85.0]
≥ 50%, n (%)	213 (68.7)	213 (74.5)
≥ 80%, n (%)	163 (52.6)	163 (57.0)
**Treatment patterns**,** n (%)**		
Remained on index LTP at end of follow-up	291 (58.3)	290 (64.6)
Discontinuation	152 (30.5)	117 (26.1)
Switch to another LTP	56 (11.2)	42 (9.4)

Abbreviations: HAE = hereditary angioedema; LTP = long-term prophylaxis; PDC = proportion of days covered; SD = standard deviation

### HAE attacks leading to ED/IP before and after LTP initiation

Among the subset of patients with > 30 days of treatment, 37.6% had ≥ 1 HAE attack leading to ED/IP during the baseline period, which numerically decreased to 22.9% during treatment (mean [median] follow-up duration: 18.2 [17.6] months; mean [median] treatment duration: 12.5 [9.3] months). The mean number of attacks numerically decreased from 0.82 PPPY during the baseline period to 0.51 PPPY during treatment (Fig. 4), most of which were treated in the ED (during the baseline period: 69.5%, during treatment: 76.5%). Among the 14.0% of patients with HAE attacks treated in an IP setting during the baseline period, the mean (median) number of IP days was 16.2 (5). The proportion of patients with HAE attacks observed in an IP setting decreased to 6.7% during treatment, with a mean (median) length of 10.5 (5.4) IP days.

### All-cause HRU before and after LTP initiation

Among the subset of patients with > 30 days of treatment, all-cause annual HRU decreased after LTP initiation but remained substantial (Fig. 5). Specifically, 15.4% of the patients had an IP admission during the baseline period with an average number of 2.4 IP days PPPY. During treatment, this proportion numerically decreased to 8.7% with an average number of 1.7 IP days PPPY. More than half of the patients (51.2%) had an ED visit during the baseline period with an average of 1.5 visits PPPY. During treatment, this proportion numerically decreased to 39.9% with an average of 1.2 visits PPPY. The average number of days with OP services was 21.0 and 19.3 days PPPY during the baseline period and during treatment, respectively.

## Discussion

This real-world retrospective study of patients with HAE shows that adherence and persistence to LTP treatment remains relatively low, with only 58.7% of patients being persistent at 12 months. Among the subset of patients who were treated with a single LTP for more than 30 days, adherence was similarly low (57.0% were adherent at 12 months), although the rates of all-cause IP admissions and ED visits descriptively declined during treatment, with the rate of HAE attacks leading to ED/IP decreasing by more than 30% compared to the year prior. Nonetheless, all-cause HRU remained substantial after the initiation of LTP, with an average of 1.7 IP days and 1.2 ED visits PPPY. While longer term data are needed to confirm these findings, they suggest that, although treatment with one of the LTP agents of interest (i.e., human C1-INH, lanadelumab, and berotralstat) can partly reduce the clinical burden of patients with HAE, there remains a need for novel therapies that may further promote better adherence and alleviate this burden.

The results of this study show that patients with HAE have suboptimal long-term adherence and persistence to LTP, even when restricting the analysis to those with more than 30 days of treatment. Patients frequently discontinued or switched LTP, and continued to experience high all-cause HRU during treatment. Some of the barriers to adherence and persistence, which may have also contributed to the high all-cause HRU, could have included the burden of frequent administrations, lifestyle incompatibility, or adverse events [10, 14–16]. For example, human C1-INH is a twice-per-week subcutaneous injection, lanadelumab is a once-every-two/four weeks subcutaneous injection, and berotralstat is a once-daily oral tablet that must be taken with food [11]. Importantly, patients with chronic conditions may prefer longer treatment intervals, and studies show that they are more likely to remain adherent when receiving their preferred dosing schedule [15, 16]. Although the adverse events associated with injectable LTP are typically mild and transient local site reactions, berotralstat can be associated with gastrointestinal symptoms (e.g., abdominal pain, diarrhea, vomiting) [14–16]. Given the chronicity of the condition, ongoing prophylactic treatment is recommended for patients with HAE to maintain disease control and reduce the frequency of HAE attacks, [17] which underscores the importance of improving the long-term treatment adherence and persistence in this population.

The current study supports the effectiveness of LTP treatment for patients with HAE in clinical practice, as demonstrated by the reduction in the rates of HAE attacks leading to ED/IP as well as the rates of all-cause IP admissions and ED visits after LTP initiation. These findings align with the results of US clinical trials and other real-world studies which have consistently demonstrated reductions in rates of HAE attacks for patients receiving LTP [18–22]. For example, the HELP OLE study, which was conducted to evaluate the long-term (≥ 30 months) effectiveness of lanadelumab, demonstrated that the treatment resulted in a 87.4% reduction in the average rate of HAE attacks [20]. Additionally, a recent claims-based analysis by Christiansen et al. showed that patients with HAE treated with berotralstat experienced a 60% reduction in HAE attack-related IP visits [22]. Given the high clinical, humanistic, and economic burden that has been previously documented among patients with HAE, [7–9] treatment with LTP also has the potential to improve other outcomes not assessed in this study by reducing the risk of HAE attacks. For instance, findings from survey studies indicated improvements in QoL, severity of the disease, work and productivity, mental health, and perceived control over illness after LTP initiation [23, 24].

While the benefit of LTP is well established, findings from the current study suggest that there remains a need for therapeutic options better suited to patient preference and lifestyle need that can promote better adherence and persistence to LTP among patients with HAE to ensure that they can derive a continued benefit from the treatment and achieve a durable reduction in disease burden. Two new LTP options have been approved by the FDA since the end of the data period included in this study. Garadacimab received approval by the US FDA in June 2025 as the first prophylactic treatment targeting factor XIIa for the prevention of HAE attacks [12]. The FDA also recently approved the first and only RNA-targeted subcutaneous prophylactic injection, donidalorsen, in August 2025 for the prevention of HAE attacks, whose low-frequency dosing schedule (i.e., every 4 or 8 weeks) may help improve treatment adherence and persistence [13]. More research is needed to better understand the real-world treatment patterns and outcomes of these novel options. In that context, the current study provides real-world evidence on the treatment patterns and outcomes associated with the currently available LTPs, establishing an important benchmark for future real-world research on emerging therapies.

## Limitations

The results of this study are subject to certain limitations. Since there is no specific ICD-10-CM diagnostic code for HAE, patients were selected based on utilization of LTP indicated for HAE. This approach could have led to the misclassification of patients with other forms of angioedema (e.g., drug-induced angioedema [including ACE-inhibitor–induced angioedema], acquired angioedema, and idiopathic non-histaminergic angioedema) treated with HAE-indicated LTP, although it is unlikely that these treatments were used off-label (even less so than ODT, given their acquisition costs). As HAE attacks cannot be directly identified in claims data, an algorithm was used to identify HAE attacks leading to ED/IP, which included ED/IP with HAE-related diagnoses. As some of these HAE-related diagnoses were non-specific (e.g., respiratory failure), it may have resulted in an overestimation of the frequency in HAE attacks leading to ED/IP. Inherent to retrospective administrative claims-based studies, the potential for coding inaccuracies in the data cannot be excluded. As patients with certain types of insurance or without health plan enrollment are not represented in the study database, the results of the study may not be generalizable to other populations and settings. This study also focused on an incident population (i.e., patients initiating a new LTP) and may not be representative of the prevalent population (i.e., patients with ongoing LTP treatments). Finally, all analyses were descriptive in nature and no statistical testing was conducted.

## Conclusions

In this real-world retrospective study, patients with HAE who initiated a recently approved LTP agent (i.e., human C1-INH, lanadelumab, and berotralstat) had fewer HAE attacks leading to ED/IP and lower all-cause HRU following treatment initiation. However, adherence and persistence to treatment were suboptimal and there remained a substantial HRU burden after treatment initiation. There is a need for novel treatment options with the potential to promote better persistence and adherence, improve patient outcomes, and further alleviate the HRU burden among patients with HAE.

## Data Availability

The data that support the findings of this study are available from Komodo Health Solutions. Restrictions apply to the availability of these data, which were used under license for this study.

## Abbreviations

C1-INH: C1 esterase inhibitor
CCI: Charlson Comorbidity Index
CI: Confidence intervals
ED: Emergency department
FDA: Food and Drug Administration
HAE: Hereditary angioedema
HIPAA: Health Insurance Portability and Accountability Act
HRU: Healthcare resource use
ICD-10-CM: International Classification of Diseases, Tenth Revision, Clinical Modification
ICER: Institute for Clinical and Economic Review
IP: Inpatient
KM: Kaplan-Meier
KRD: Komodo Research Database
LTP: Long-term prophylaxis
ODT: On-demand treatment
OP: Outpatient
PDC: Proportion of days covered
PPPY: Per patient per year
SD: Standard deviation
QoL: Quality of life
US: United States
